# Use of Vegetable Fibers for PRB to Remove Heavy Metals from Contaminated Aquifers—Comparisons among Cabuya Fibers, Broom Fibers and ZVI

**DOI:** 10.3390/ijerph14070684

**Published:** 2017-06-24

**Authors:** Celia Margarita Mayacela Rojas, María Fernanda Rivera Velásquez, Adalgisa Tavolaro, Antonio Molinari, Carmine Fallico

**Affiliations:** 1 Department of Civil Engineering, University of Calabria, Rende (CS) 87036, Italy; margaritamayacela@hotmail.com (C.M.M.R.); ant.molinari2002@libero.it (A.M.); 2 Faculty of Engineering, National University of Chimborazo, Riobamba EC060104, Ecuador; mafer.rivera@live.com; 3 National Research Council (C.N.R.-I.T.M.), Rende (CS) 87036, Italy; a.tavolaro@itm.cnr.it

**Keywords:** aquifer contamination, heavy metals, human health, permeable reactive barriers, remediation, vegetal fibers

## Abstract

The Zero Valent Iron (ZVI) is the material most commonly used for permeable reactive barriers (PRB). For technical and economic reasons, hoter reactive substances usable in alternative to ZVI are investigated. The present study takes into account a vegetable fibers, the cabuya, investigating its capacity to retain heavy metals. The capacity of the cabuya fibers to adsorb heavy metals was verified in laboratory, by batch and column tests. The batch tests were carried out with cabuya and ZVI, using copper (Cu), zinc (Zn), cadmium (Cd) and lead (Pb). The results obtained by the cabuya fibers showed a very high adsorption capacity of heavy metals and resulted very similar to those obtained for the broom fibers in a previous study. The high value of the absorption capacity of the cabuya fibers was also confirmed by the analogous comparison made with the results of the batch tests carried out with ZVI. Column tests, using copper, zinc and cadmium, allowed to determine for the cabuya fibers the maximum removal percentage of the heavy metals considered, the corresponding times and the time ranges of the release phase. For each metal considered, for a given length and three different times, the constant of degradation of cabuya fibers was determined, obtaining values very close to those reported for broom fibers. The scalar behavior of heavy metal removal percentage was verified. An electron microscope analysis allowed to compare, by SEM images, the characteristics of the cabuya and broom fibers. Finally, to investigate the chemical structure of cabuya and broom fibers, the FTIR technique was used, obtaining their respective infrared spectra.

## 1. Introduction

Groundwater pollution is increasingly widespread because of human activities, mainly related to agricultural practices and industrial activity. In many cases, pollution is due to the presence of heavy metals, which, often abandoned or stored on the soil surface without any expedient, infiltrate into the ground and reach the underlying aquifer, constituting a real hazard for human health. Therefore, remediation of contaminated aquifers is necessary more and more frequently [1,2,3,4].

Regarding the contaminated aquifers remediation, there are many techniques expounded in the literature, each of these presents a more or less high level of efficiency, in terms of contaminant removal, and different costs, although always high [1,4,5,6,7,8,9,10] The remediation technique to be used should be chosen case by case, according to the characteristics of the aquifer, site and type of contaminant which must be removed. Among the various reclamation techniques of aquifers, reference is made in this study to the use of Permeable Reactive Barrier (PRB), which is a passive technique widely used for in situ remediation, and is often cheaper than other techniques, such as pump and treat, This method gained popularity, because of its efficiency in removal of pollutants and owing to its limited costs [11,12,13]. The PRB technique is relatively simple: the reactive material is placed in the ground to form a barrier that intercepts the contaminant plume that moves thanks to a natural gradient. Groundwater passes through the reactive medium, the physical and chemical processes occurring inside the barrier eliminate or transform contaminants into less harmful or not moving species [14,15,16,17,18,19,20,21,22]. There are numerous reactive materials that can be used for a permeable barrier. Very often the cost of the reagent materials most commonly used for the construction of the PRB is high and their availability is not immediate [6]. With reference to pollution by heavy metals, among the materials that can be used to build a PRB, the most common is certainly ZVI, because of its availability and affordability [19,23,24,25,26,27]. However, ZVI has some negative aspects, such as the formation of products of corrosion and various substances in solid phase that precipitate and accumulate on the bottom of the PRB, often causing, over time, a decrease in porosity and permeability, with consequent tendency to reduce the efficiency of the barrier [28,29,30,31]. This prompted many researchers to investigate the reactive capacity of several materials that could be used in place of zero valent iron, at an accessible cost, in an effort to increase the knowledge of their characteristics and potential [32,33,34,35,36].

This paper considers a readily available vegetable substances, cabuya (Furcraea Andina). The reactive properties of this substance for some of the most common heavy metals, namely Copper (Cu), Zinc (Zn), Lead (Pb) and Cadmium (Cd), were appropriately investigated in the laboratory in two successive steps. In the first step, the adsorption capacity of the cabuya for the heavy metals considered, was verified by static batch tests. In the second step, dynamic column tests were carried out, taking into account samples of vegetable fiber investigated, in order to verify the adsorption time, to detect the time in which the beginning of the release occurs and to characterize the manner in which such phenomena occur. Finally, with regard to the toxic substances considered, comparisons were performed between the behavior of the cabuya fibers and those of another vegetable substance, the Spanish broom (Spartium junceum), for which the results of a similar investigation can be found in a previous study [36]. Comparisons were also made with ZVI, for which similar laboratory investigations were carried out, so as to have a homogeneous comparison of the parameters.

## 2. Cabuya Fibers

Vegetable fibers are used more and more frequently for the treatment of contaminated water [4,33,35,36,37,38,39,40,41,42,43,44,45,46,47,48,49,50,51,52]. In fact, the polymeric structure of these fibers, containing cellulose, hemicellulose, pectin, lignin and protein, has a remarkable ability for ion exchange that gives to the fibers a high adsorption power against many substances [38,39,40,42,43,53,54]. Thus, the lignocellulosic fibers act on the contaminated waters as a real filter which retains the pollutants, specifically heavy metals [38,55,56] The large lignin molecules, composed of functional groups linked to each other and lacking stereo-regularity, fill three dimensions, often forming a gummy mixture of lignins with a wide range of molecular weights, according to the plant species and sample history [53,57,58]. In general, the molecular weight of lignin varies from 2000 g/mol to 15,000 g/mol [37,42]. Moreover, it is not soluble in water, has a strong resistance to chemical reactions and a large surface area (180 m^2^/g) [42]. These features reveal that the lignin is a means with a good potential of adsorption, which can be used to remove heavy metals from wastewater [59]. It is evident that the adsorption action of pollutants, specifically of heavy metals, is greater the higher the contact surface between the contaminated water and the walls of the fibers containing lignin. Therefore, the density of the fibers and their dimensions, specifically their diameter, are crucial for the effective adsorption of the pollutant [36]. Generally, the raw fibers are obtained with simple carding, aiming to lay bare the core of the stem, namely its inner part, which is usually the one containing the highest percentage of lignin [58] The fibers considered in this paper are those of a very common vegetable species, the cabuya (*Furcraea andina*) or fique plant, which is a leaf fiber. In the following, the fibers of this substance will be always considered in its raw state, namely subjected to a simple treatment that provides for the decortication and carding, followed by cleaning with pressure washers and subsequent drying.

This plant belongs to the class of leaf succulents, specifically to the trees of the order of Agavales. It is a native of the entire Andean regions, Central America and the Caribbean area (Ecuador, Colombia, Mexico, Perù, etc.) and it grows easily in the wild or can be cultivated in valleys and on hillsides. This plant has a small but strong stalk and sword-shaped rigid leaves with thorns at the edges [60,61]. Substantial changes in the diameter of the fiber can be detected from plant to plant and along the same fiber, but generally these do not exceed 400 μm [60,61,62]. The cellulose content of the fibers can vary between 18.7% and 70%, while that of the lignin is generally variable between 6.81% and 15.5% [62,63]. Cabuya fibers are very durable and are employed for many industrial uses (buildings, components, thermal and acoustic insulation, etc.), as well as reactive substances for the adsorption of pollutants in the treatment of contaminated water [60,61,62,63,64]. However, it must also take into account that the lignin can be subject to degradation by microorganisms, white-rot fungi, which produce particular enzymes termed “ligninases” [65,66,67]. The consequent impact on the environment can be very negative, but these aspects require specific investigations that are beyond the scope of this study.

## 3. Methods

**Figure 1:**
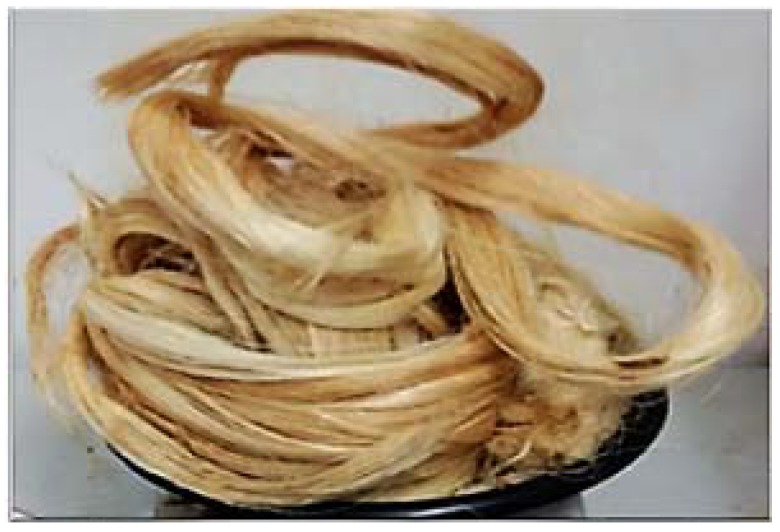
Sample of already treated cabuya fibers.

Initially, a suitable amount of cabuya fibers was prepared, extracting them manually and performing the decortication treatments, carding, cleaning with a pressure washer and drying. Figure 1 shows some samples of cabuya fibers obtained by the simple treatment described above.

However, the experimental activity in the context of the present study was carried out on pieces of fibers, obtained by cutting the cabuya fibers so as to obtain parts with a length of about 10 cm. This expedient was made both to improve the contact between fiber and pollutant, and in an attempt to avoid clumps and clusters of fibers that could also change the flow conditions of contaminated water within the PRB. To verify the efficiency of cabuya fibers in purifying groundwater containing heavy metals, a series of laboratory tests were conducted.

Moreover, cabuya fibers were analyzed by Scanning Electron Microscope (SEM), which uses a focused beam of high-energy electrons to generate a variety of signals at the surface of solid specimens. The signals derived from electron-sample interactions reveal information about the sample, including external morphology (texture), chemical composition, and crystalline structure and orientation of materials making up the sample.

This technique is the most powerful tool to investigate the chemical structure of natural fibers [68]. By this technique, spectra were directly collected from the fiber surfaces over different points of the sampling area at the same pressure with a micrometer torque. This allowed also to obtain the infrared spectrum of cabuya fibers. This analysis was also performed here for broom fibers. In addition, the Fourier transform infrared spectrometry (FTIR) technique was applied, providing information on the structures of the main constituent species of both the vegetable fibers investigated [69].

### 3.1. Hydraulic Characterization

For the aims of this study the hydraulic characterization of the fibers considered is very important. In fact, these, having to constitute the body of the PRB within the saturated aquifer, must ensure the barrier will really act like a filter, retaining pollutants and allowing water passage. Therefore, the PRB must have a hydraulic conductivity (*k*) greater than that of the surrounding porous medium, in which it was inserted otherwise the contaminated water of the aquifer, instead of being attracted towards this, perceives the barrier as an obstacle and tends to circumvent it.

**Figure 2:**
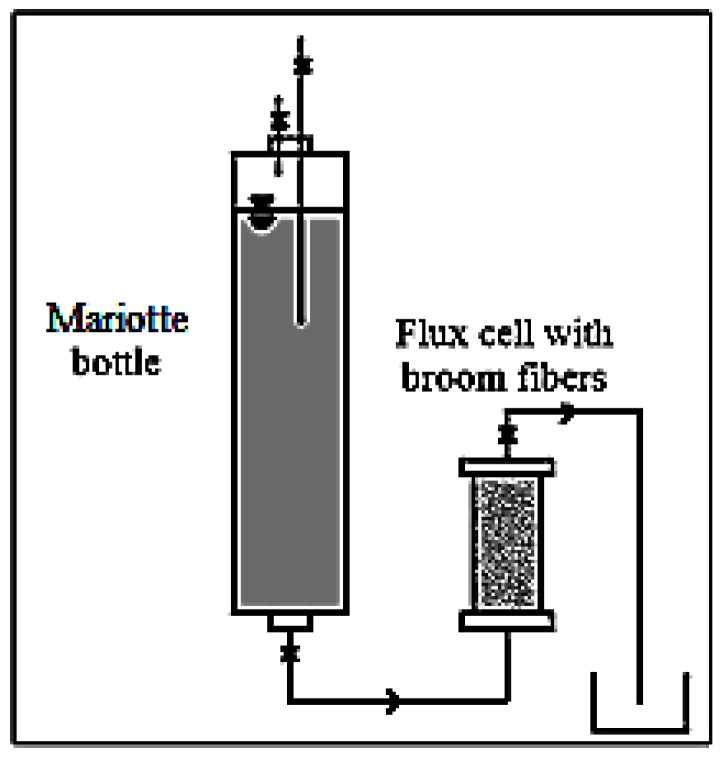
Scheme of device used to measure permeability.

The hydraulic conductivity measurements were carried out in the laboratory, using a flow cell working as a permeameter at constant head. The scheme of the device utilized is shown in Figure 2.

The sample, consisting of the specific fibers with the assigned density, was placed in a Plexiglas cell, which had a porous membrane at each end to allow the water flow. The water inlet was provided by a plastic tube connecting a Mariotte bottle to the bottom of the cell, while the water outlet was at the top of it. The plastic tube linking the Mariotte bottle and the cell has also the role of maintaining a constant hydraulic head. In this way, the air contained in the sample can be removed. The cylindrical cell is 0.60 m long and has a diameter of 0.064 m. The density of all the fiber samples examined was set at 160 kg/m^3^. The hydraulic conductivity (*k*) was measured on saturated samples at hydraulic heads between 0.34 m and 0.64 m and for each sample the measurement was repeated three times [70]. Moreover, the effective porosity, considered as the ratio between the water volume drained by gravity and the total volume of the saturated sample, for the density considered, was also measured. The saturated sample was initially weighed, then placed on a special grid, so that it was drained by gravity. Later, when the dripping was finished, the sample was weighed again. The difference between initial and final weight represented the weight of drained water by gravity. Knowing the water density, it was possible to determine the volume of water drained, which, divided by the total volume of the saturated sample, provides the effective porosity that contributed effectively to the water flow within the considered medium [71,72,73,74].

### 3.2. Batch Tests

Batch tests are commonly carried out to verify the reactivity of a substance with regard to a specific pollutant. The removal efficiency of the substance considered, in terms of adsorption capacity, is checked by making the substance remain static while in contact with a solution containing an assigned concentration of the pollutant, for a more or less long time. In this way, the residual concentration of pollutant and the percentage of adsorption can be verified in the sampling times.

To verify the adsorption capacity of cabuya fibers with regard to the metal cations of copper (Cu), zinc (Zn), cadmium (Cd) and lead (Pb) in aqueous solution, specific batch tests were carried out for each of the heavy metals considered. Therefore, 300 mg of fibers were placed in a glass container in contact with an aqueous solution of known heavy metal concentration. These concentrations were set assuming an amount not less than 10 times the corresponding limit value for each pollutant according to Italian regulations. Table 1 shows the concentrations values utilized for the batch tests for each of the heavy metals considered and the corresponding values set by the Italian regulations [75]. Because of the higher frequency of nitrate ions in soils, it was decided to use the nitrates for the solutions of these metals: cadmium nitrate tetra-hydrate [Cd(NO_3_)_2_4H_2_O], copper nitrate tri-hydrate [Cu(NO_3_)_2_3H_2_O], lead nitrate [Pb(NO_3_)_2_], zinc nitrate hexa-hydrate [Zn(NO_3_)_2_6H_2_O]. The closed glass containers were mixed vigorously and continuously on a shaker under ambient conditions for each test. After a maximum time T of 30 h, the solution samples were analyzed using an ICP-MS instrument, based on inductively coupled plasma mass spectrometry which is capable of detecting metals and several non-metals at very low concentrations (ppt or ng/L) and with great speed, precision and sensitivity. In this way, for each batch test, the value of the final concentration of the heavy metals considered and the corresponding adsorption degree of the fibers were verified.

### 3.3. Column Tests

**Figure 3:**
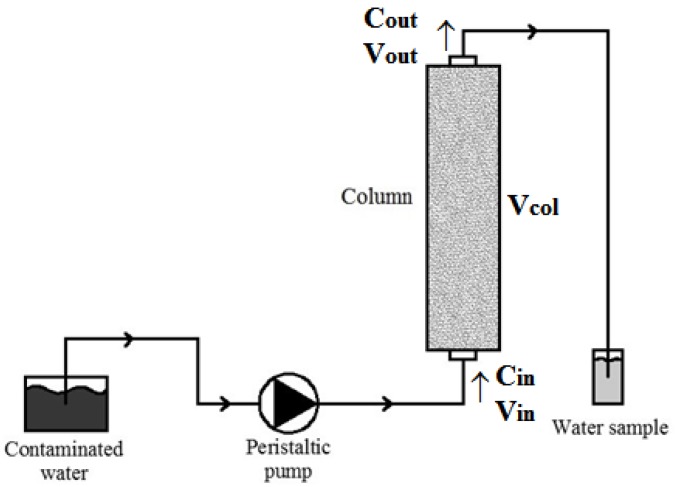
Schematic representation of the experimental device used for the column test.

Batch tests require little time, but the column tests, conducted continuously, are able to determine the process parameters under dynamic flow conditions and therefore in conditions closer to real ones, expected in a PRB system built in the field [27,76]. Thus, column tests provide the best indications of the optimal ratios for cost-effectively removing contaminants while maintaining satisfactory hydraulic conductivity. Therefore, column tests were performed to verify the removal efficiency of the pollutants considered by the cabuya fibers during the hydraulic drainage within the column. For each fiber sample on which the test was performed it was verified that the structure, texture, location and degree of compaction were compatible with the water flow in the aquifer. Moreover, it was verified for each test that the velocity value was such as to ensure an adequate contact time between the contaminated solution and the medium reagent, namely the fibers, to ensure a complete process of detention and degradation of the contaminant. The tests were conducted and repeated with 15, 30 and 60 cm long Plexiglas columns with an internal diameter of 4.4 cm, filled with the fiber samples with a density of 160 kg/m^3^. The column tests were carried out using only copper, zinc and cadmium as pollutants, as, for reasons of laboratory management, it was not possible to perform the column tests for lead. The flow of the solution through the column, with the assigned concentrations of pollutants reported in Table 1, was provided continuously by a peristaltic pump set at 5 r/min (≈10 m/day) in a way that still has a speed plausibly close to the normal rate of filtration in the groundwater. The flow was set from the bottom to the top of the column by allowing the escape of any air bubbles. At the top of the column, where there is the water output, samples were taken periodically and subjected to concentration measurement. The column test apparatus is sketched in Figure 3.

It is possible to obtain the removal percentage of single contaminant as:

(1) $$removal\;\%=\frac{C_{in}\times V_{in}-C_{out}\left(V_{col}+V_{out}\right)}{C_{in}\times V_{in}}\times 100$$

where *C_in_* represents the initial concentration (ML^−3^), *V_in_* the initial volume (L^3^), *C_out_* the final concentration (ML^−3^), *V_col_* the retained volume in the column (L^3^) and *V_out_* the output volume from the column (L^3^).

This type of test is essential for a correct dimensioning of a PRB, as it allows the kinetics of degradation of the pollutant considered, and then the thickness of the barrier to be defined. Indeed, the optimal thickness of reactive materials in a PRB is a trade-off between maximizing the removal efficiency and longevity and minimizing construction costs, namely minimum dimensions and cost of the reactive material [77,78,79]. In any case, it is advisable to determine the minimum thickness of reactive materials for a PRB longevity of not less than 10 years.

### 3.4. Kinetic of Degradation

The effectiveness of the cabuya fibers in the treatment of heavy metals can be verified determining the constant of degradation (*λ*). This parameter allows the concentration of the contaminant to be linked, as heavy metals, with the change in concentration over time. A first-order kinetics, useful to size the PRB’s, describes this variation [19,80] by the following equation:

(2) $$\frac{dC}{dt}=-\lambda \rho_{m}C$$

where *C* is the heavy metals concentration at *t* time (ML^−3^), *λ* the constant of degradation (L^3^M^−1^T^−1^), *dC*/*dt* the removal rate (ML^−3^T^−1^) and *ρ_m_* the metal density (ML^−3^). The experimental tests allow the constant of degradation (*λ*) to be determined for each metal by the following equation, once the thickness of the PRB is fixed:

(3) $$\lambda =\frac{ki}{S\rho_{b}}ln\left(\frac{C_{0}}{C}\right)$$

where *ρ_b_* is the bulk density (ML^−3^), *C_0_* the initial concentration (ML^−3^), *k* the hydraulic conductivity (permeability) (LT^−1^), *i* the hydraulic gradient, *C* and *λ* have the meaning above specified and *S* is the thickness of the PRB (L), which can be determined by the following equation:

(4) $$S=\frac{V}{A}=\frac{ki}{\lambda \rho_{b}}ln\left(\frac{C_{0}}{C}\right)$$

where *V* is the volume of the PRB (L^3^), *A* the corresponding area (L^2^) and the other symbols have the meaning already specified above. The Equations (3) and (4) can be used to size the PRB.

## 4. Results and Discussion

Each of the hydraulic conductivity measurements, carried out to characterize the cabuya fibers, was repeated three times, with a fiber density of 160 kg/m^3^ and constant hydraulic head of 30 cm, allowing the following mean value to be obtained: *k* = 4.95 × 10^−4^ m/s. Although the value of *k* obtained for the cabuya fibers is slightly greater than that determined by Fallico et al. [33] for the broom fibers (*k* = 1.72 × 10^−4^ m/s), it should be emphasized that the order of magnitude of this parameter remains substantially the same for both these fibers. However, the difference, albeit limited, of the constant hydraulic head on which the measurement tests were conducted in the two cases (30 cm for the cabuya fibers and 25–50 cm for the broom fibers) allows purely indicative comparisons to be made. Furthermore, for the assigned value of density, the effective porosity was 0.54, the average value of pH was 6.71 and the content of lignin equal to 11.30%. To verify the removal capacity by cabuya fibers for the heavy metals considered (Cu, Zn, Cd and Pb), a specific batch tests was carried out according to the procedures described above, using solutions with initial concentration values of the pollutants given in Table 1. The maximum values of removal percentage, residual concentration and corresponding contact time are shown in Table 2. In order to make a reliable comparison with ZVI, the results of similar batch tests, carried out with this substance, are also reported in the same Table 1, considering the same amount of ZVI used for cabuya and solutions with the same concentrations of heavy metals contained in Table 1.

**Figure 4:**
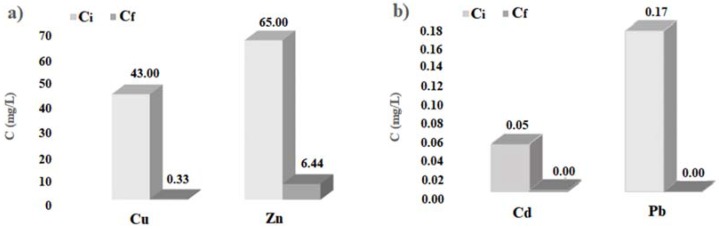
Initial (C_i_) and final (C_f_) concentrations of the batch tests carried out with cabuya fibers. (**a**): for Cu and Zn; (**b**): for Cd and Pb.

**Figure 5:**
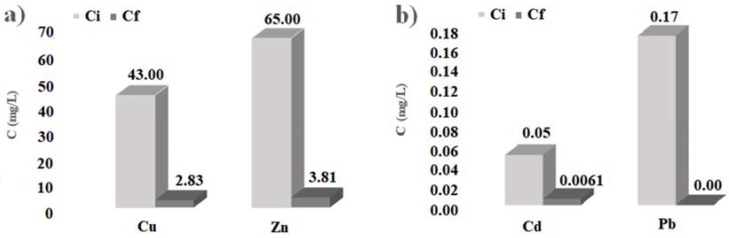
Initial (C_i_) and final (C_f_) concentrations of the batch tests carried out with ZVI. (**a**): for Cu and Zn; (**b**): for Cd and Pb.

The histograms of Figure 4 show the initial and final concentration of heavy metals considered, obtained by the batch tests carried out with cabuya fibers. Analogously, the histograms of Figure 5 show the initial and final concentration of heavy metals considered, obtained by the batch tests carried out with ZVI.

**Figure 6:**
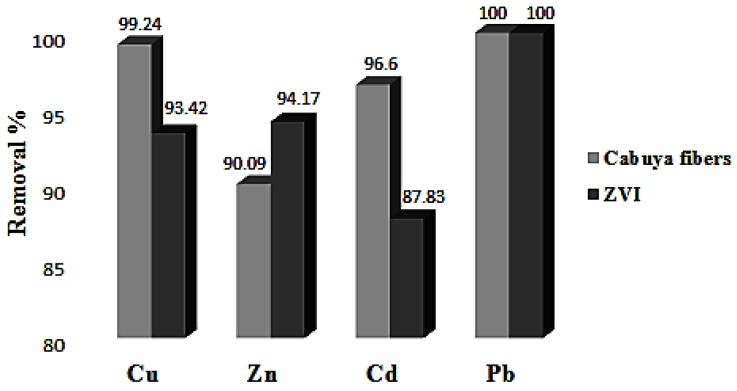
Removal percentages of heavy metals considered for cabuya using ZVI.

Furthermore, in Figure 6 the histograms of removal percentage of heavy metals considered, for cabuya and ZVI, are reported.

The results of the batch tests, carried out with the broom from Fallico et al. [36] showed adsorption values of 51.6% for cadmium, 73.3% for lead and 72.2% for zinc, which, however, also in this case, cannot be compared with those obtained for the cabuya in this paper because of the different conditions with which the tests were performed.

In any case, the results obtained here allow affirmation that also the cabuya fibers are reactive towards the heavy metals considered, presenting high percentages of pollutant removal, particularly for copper, cadmium and lead, as evidenced by the values given in Table 2 and the histograms of Figure 4.

The values of the parameters given in Table 2 and the histograms of Figure 4, Figure 5 and Figure 6 show that the cabuya fibers are reactive with respect to the heavy metals considered just as ZVI. Specifically, the removal percentage values of cabuya show that these fibers are slightly more reactive than ZVI with regard to copper and cadmium, while they are slightly less reactive with regard to zinc and exhibit the same reactivity with regard to lead. Moreover, for ZVI the contact times, corresponding to the removal percentages given above, are generally greater than or equal to those determined for cabuya, except for zinc.

In accordance with the positive results obtained with the batch tests, column tests were carried out, in order to obtain an objective response about the effectiveness of treatment by filtration through the cabuya fibers and analyzing the values of contaminant concentrations at the end of the filtration process. The results of the column tests, performed using copper, zinc and cadmium as pollutants, are reported, for the different lengths of the considered column, in Table 3.

**Figure 7:**
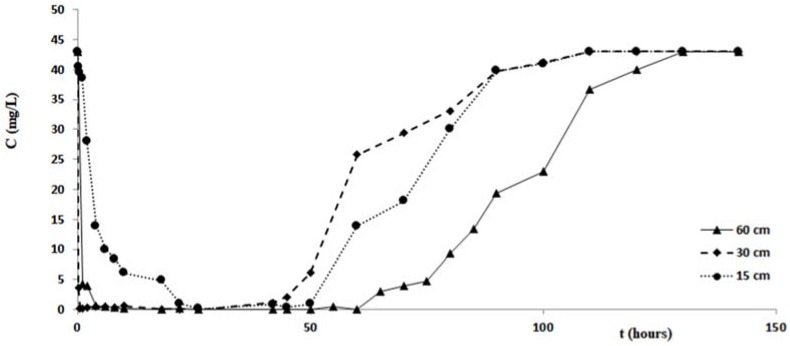
Variation of copper concentration vs. time for the tests with column length equal to 15 cm, 30 cm and 60 cm.

**Figure 8:**
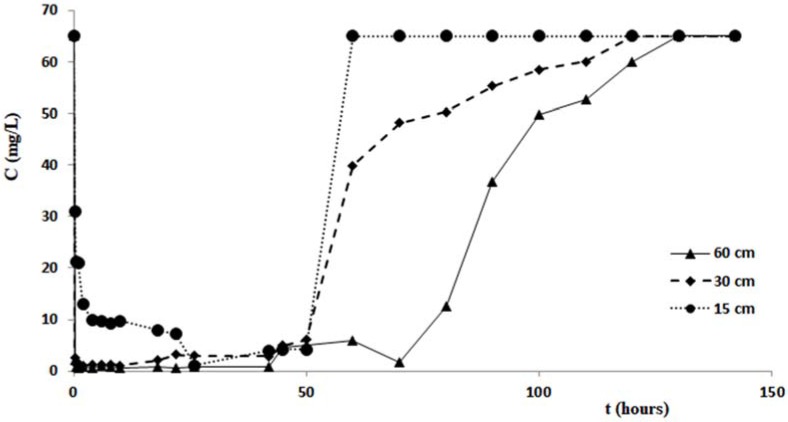
Variation of zinc concentration vs. time for the tests with column length equal to 15 cm, 30 cm and 60 cm.

**Figure 9:**
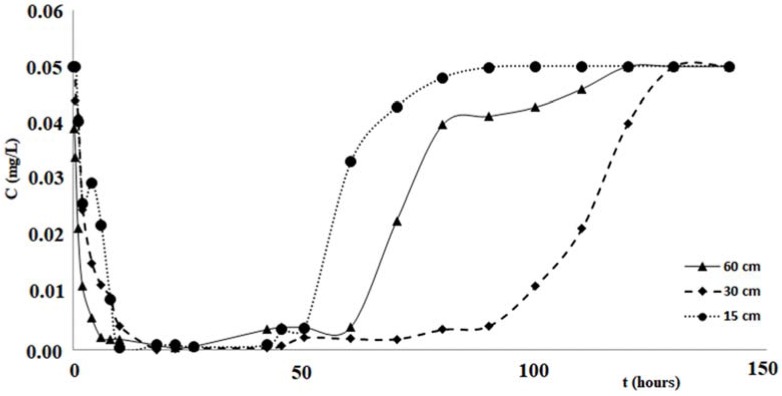
Variation of cadmium concentration vs. time for the tests with column length equal to 15 cm, 30 cm and 60 cm.

The duration of each test was 150 h, in order to investigate not only on the process of removing the pollutant, but also the next step of release. For the duration of each test, at fixed times, appropriate sample outputs from the columns were collected, then subjected to analysis to determine the minimum value of the concentration, the corresponding value of the maximum removal percentage, the filtration times corresponding to these maximum values and the duration of the pollutant release step. Figure 7 shows the variations of the copper concentration over time, for each test performed with the columns of different length. Analogous changes in concentrations of zinc and cadmium are shown in the graphs of Figure 8 and Figure 9. Table 3 and Figure 7, Figure 8 and Figure 9 confirm that the cabuya fibers have a high removal capacity of the heavy metals investigated. This is certainly true for copper, although also for zinc and cadmium the removal recorded in the column tests was almost total. The maximum value of the removal percentage was obtained for copper after 26 h, for all three columns of different lengths. For zinc these times vary from 2 to 26 h, according to the length of the column, while for cadmium these vary from 10 to 22 h in a manner increasing with the length of the column.

Specifically, for the zinc we observe that the filtration time, corresponding to reaching the minimum concentration value, was at the maximum for the test carried out with the column length equal to 15 cm, while for columns of length 30 cm and 60 cm this time increased with length. This result did not correspond to ourexpectations, and the causes are probably due to a malfunction of the measurement apparatus.

**Figure 10:**
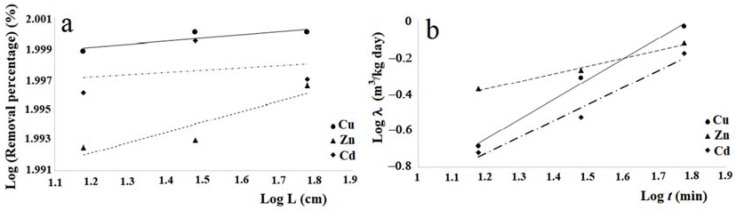
(**a**) Scalar behaviour of heavy metal removal percentage vs. column lenght (L). (**b**) Trends of experimental laws λ=λ(t) for Cu, Zn and Cd.

If one compares the removal percentages obtained in the tests carried out with the three columns of different lengths, it is possible to notice that the lower percentage is obtained with the column of length 15 cm, while it seems possible to observe an increase of this removal percentage when the column length increases. This circumstance, more pronounced for the tests performed with zinc, could be due to less contact time between the pollutant and fibers relative to the column of 15 cm. This hypothesis seems to be verified by tests performed with cadmium, posing filtration times, relative to the maximum value of removal, increasing with the length of the column, while for copper such filtration times relative to Cout (min) are the same for all three of the columns and lengths considered for the zinc values of the times of filtration without any correlation with the length of the columns. This hypothesis seems to be verified by tests performed with cadmium, which present filtration times, relative to the maximum value of removal, increasing with the length of the column, while for copper such filtration times relative to Cout (min) are the same for all three column lengths considered, and for the zinc values of the filtration times without any correlation with the length of the columns. As regards the step of pollutant release, the data of Table 3 and the graphs of Figure 7, Figure 8 and Figure 9 indicate different durations, according to the pollutant considered and the length of the column. Specifically, for copper the shortest duration (about 90 h) was found for the test carried out with the column of 60 cm in length, while for those of 15 cm and 30 cm durations respectively of about 105 h and 124 h were found. For zinc the minimum duration of the pollutant release step was 108 h for the test with the 60 cm length column with the maximum of 142 h for the test with the 30 cm length column, while for the test with the 15 cm length column the release duration was 124 h. For cadmium the maximum duration of the release, 124 h, was verified for the test with the 60 cm length column, while for those of 15 cm and 30 cm the same duration was found, of about 108 h. The results obtained demonstrated that in many cases, once the maximum percentage of the pollutant removal was reached, meaning the minimum value of its concentration, the permanence of these conditions occurs for a more or less ample time before the beginning of the pollutant release step. The tests performed by columns of different length, allowed a scalar behavior of the percentage removal of pollutants to be detected, as Figure 10a in logarithmic scale shows. In fact, this graph shows the removal percentages trends, of copper, zinc and cadmium respectively vs. L, which represents the length in centimeters of the column used for the tests.

The laws adopted to represent the scalar behavior are of power, that is:

*removal percentage* (%) = *a*·L^*b*^ (5)

where the parameters *a* and *b* that define the Equation (5) respectively for the three heavy metals considered are given in Table 4. The values of the determination coefficients R^2^ relative to the Equation (5) for the heavy metals considered, show that the equations determined for copper and zinc are sufficiently meaningful, while that for cadmium can be considered insignificant. Moreover, when one compares the removal percentage of copper, zinc and cadmium with regard to the cabuya fibers given in Table 3 with those obtained by Fallico et al. [36] by column tests carried out with raw broom fibers for the same pollutants, one notes that there is a substantial correspondence.

However, the removal times obtained for the raw broom fibers in the investigation of Fallico et al. [36], resulting equal to about 1 h, are considerably lower than those obtained in the present paper with cabuya fibers. The values of the constant of degradation (*λ*) were determined using Equation (3) for the tests performed with the 60 cm length columns, for each metal considered and for times of 15 min, 30 min and 60 min. For this purpose, the thickness (*S*) of the barrier in the direction of flow equal to 0.6 m was assumed, the bulk density (*ρ_b_*) was set equal to the density of the broom fibers in the column, namely 160 kg/m^3^, the hydraulic conductivity (*k*) had the value derived from the hydraulic conductivity test, the gradient (*i*) was determined for each test, once the corresponding values of the filtration speed, the values of the initial concentrations and that obtained at the exit of column in the times considered were known. These values of the constant of degradation are shown in Table 5.

Considering the values of *λ* at 1 h, it can be seen that for copper and zinc the values determined for the cabuya are very close to those reported for Spanish broom in the study of Fallico et al. [36]. For cadmium this value is lower than that obtained for Spanish broom in the study cited. Altogether it can be observed that the values of *λ* determined for the three heavy metals considered grow with increasing time. Even in this case, the correlation between *λ* and the time (*t*) can be described by a power law, similar to the Equation (5), given by:

(6) $$\lambda =c\times t^{d}$$

which is defined, respectively for the three pollutants considered, for the values of the parameters *c* and *d* shown in Table 6.

The trends of the experimental laws λ=λ(t), respectively for copper, zinc and cadmium, are shown in the Figure 10b. The representation of the laws *λ* = *λ* (*t*), given in Figure 10b, demonstrates the increasing trend of *λ* with *t*.

Furthermore, analyzes of cabuya fibers were carried out with a scanning electron microscope which allowed the size of fiber bundles (technical fibers) to be determined.

**Figure 11:**
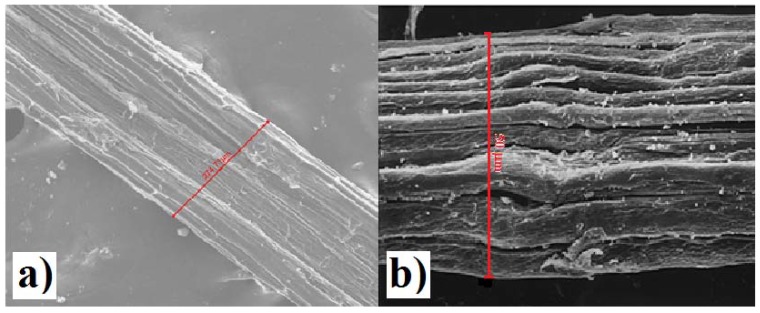
SEM image for bundles of: (**a**) cabuya fibers; (**b**) broom fibers, including information on their size.

Figure 11 shows SEM images of fiber bundles of cabuya (Figure 11a) and Spanish broom (Figure 11b) respectively.

Electron microscope analysis showed that bundles of cabuya fibers tend to assume larger diameters than those of Spanish broom, as evidenced by a comparison of SEM images Figure 11a diameter equal to 274 μm and Figure 11b diameter equal to 50 μm.

**Figure 12:**
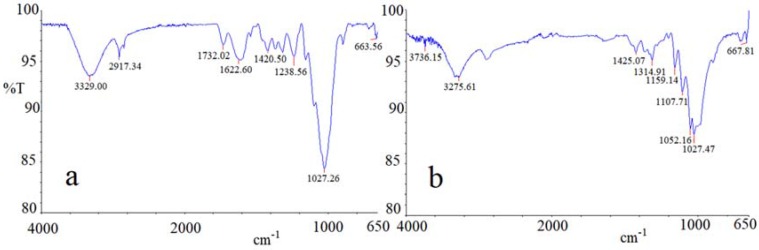
(**a**) Infrared spectrum of cabuya fibers obtained by FTIR technique. (**b**) Infrared spectrum of broom fibers obtained by FTIR technique.

To investigate better the mechanisms that contributed to determine the behaviors highlighted and the results obtained, the FTIR (Fourier transform infrared spectroscopy) technique was used for both cabuya and broom fibers. Figure 12a,b show the infrared spectrum of cabuya and broom fibers, respectively. The graphs a and b of Figure 12 have the wave number in abscissa, expressed in cm^−1^, while in ordinates have the transmittance (T), namely the ratio of the intensity of the transmitted light to the intensity of the incident light, expressed in percent [81].

The spectral profiles of two fibers are rather similar, indicating similar structures of the main constituent species (cellulose, hemicellulose and lignin). Nevertheless, a difference between the vibrational spectra can be noted as a result of the increased presence of lignin in cabuya. This can be speculated due to the presence of two distinct adsorption medium bands at 1622 cm^−1^ and 1732 cm^−1^ corresponding to C=C vibrational bending and to the acetyl and uronic ester groups of ester linkage of the ferulic and p-coumaric acids of lignin, respectively [82]. Given that lignin connects the elementary fibers, a greater presence of this substance in cabuya fibers than in those of broom could justify the larger size of bundles of fibrous cabuya than those of the Spanish broom. Furthermore, the presence of a strong band at around 3329 cm^−1^ can be observed, which is attributed to different O–H stretching modes, and another two bands at around 2917 cm^−1^ and 2850 cm^−1^, related to asymmetric and symmetric methyl and methylene stretching groups present in all the fiber spectra, but most notably in the cabuya spectrum. The very strong vibrational bands centered at 1027 cm^−1^, which is characteristic of cellulosic natural fibers, comprises, as included bands, the adsorptions of C–O–C asymmetric stretching (1152 cm^−1^), C–C, C–OH, C–H ring and side group deformations (1046 cm^−1^ and 1027 cm^−1^). In the fingerprint region, four medium bands can be observed: the band centered at 1423 cm^−1^, which is attributed to HCH and OCH in-plane bending; the adsorption at around 1370 cm^−1^, which is related to in-the-plane CH bending, the band at 1318 cm^−1^, which is due to CH_2_ rocking vibration at C6 and to the S ring stretching; finally, the band centered at 1238 cm^−1^, which can be attributed to C–C, C–O, C=O stretching and to the COH bending at C6. Finally, the weak vibrational band centered at 663 cm^−1^ can be related to the C–OH out-of-plane bending mode.

## 5. Conclusions

The technique of Permeable Reactive Barriers is more and more frequently used, for its economic advantages and reduction of the time required for remediation compared to other methodologies, such as, for example, that of the pump and treat. The most frequently used material for building of the PRB is certainly ZVI, which however has some drawbacks, owing to the formation of compounds which tend to precipitate on the bottom of the barrier and which, in time, end up reducing its hydraulic conductivity. This has led many scientists to search for innovative substances that can advantageously replace ZVI in the creation of the PRB. Among these substances are also included some vegetable fibers and the present study reports the results obtained by investigating the adsorption capacity of certain heavy metals by cabuya fibers in the raw state. Once the hydraulic characterization of these fibers is carried out, determining their hydraulic conductivity and porosity, a series of batch tests, with solutions of copper, zinc, cadmium and lead, was performed. These tests showed a high adsorption capacity of the cabuya fibers for the heavy metals considered. Similar batch tests were repeated using ZVI instead of cabuya, with the same heavy metals (Cu, Zn, Cd and Pb), obtaining results completely comparable with those obtained for cabuya.

Afterwards, to test the removal capacity of pollutants by the cabuya, only for this substance column tests were conducted using contaminating elements such as Cu, Zn and Cd and columns of different length (15 cm, 30 cm, 60 cm). These tests allowed to determine the removal percentages of pollutants in flow conditions close to the real ones and for columns of different lengths. The values obtained were very high for all three heavy metals considered, together with the variations of concentration in time and the durations of the steps of adsorption and release of pollutants. The results obtained show that in most cases the duration of the adsoption step is significantly limited compared to that of release. Moreover, it was observed that, in most of the tests carried out, between the adsorption step and the subsequent one of release, a permanence of the pollutant concentration around the minimum value occurred, in some cases very evident. These results allowed detection of a scaling behavior of the removal capability of the cabuya with respect to the heavy metals considered, allowing the experimental scaling law to be determined for each of these pollutants. Furthermore, the results of the tests carried out with the 60 cm length columns allowed the values of the constant of degradation (*λ*) to be determined for each heavy metal considered and for fixed times. Therefore, an approximate prediction of the behavior of the fiber in adsorption of each of the contaminants analyzed, owing to different chemical and physical conditions in the groundwater, could be made. Specifically, on the basis of the *λ* values to 60 min, it is conceivable that copper, which is characterized by a higher value of *λ*, needs less time to degrade, while cadmium takes more time. An increasing trend of *λ* for growing times was also found for each pollutant. The results obtained here were compared with those obtained by Fallico et al. [36] for broom fibers, verifying substantially similar behavior. In fact, also in all cases analyzed here a high removal efficiency, with a significant reduction of the concentration of single pollutants, was obtained, in some cases very close to 100%. Finally, the analysis at the scanning electron microscope, carried out on both cabuya and broom fibers, allowed significant information on the actual dimensions of the relative fiber bundles to be obtained. In this regard, the FTIR technique was used to investigate the chemical structure of the two fibers examined, obtaining rather similar spectral profiles for both and an increased presence of lignin in the cabuya.

This investigation highlighted the high removal capacity of the cabuya fibers for the heavy metals considered. These fibers, as also those of Spanish broom, investigated previously [36], give similar results to those of ZVI. Therefore, for the construction of the PRB, considering the ready availability of the plants taken into consideration, the simplicity of the treatment to obtain the raw fibers, the resulting low cost and the absence of drawbacks that characterize the use of ZVI, it seems justified to consider the use of these vegetable fibers advantageous instead of ZVI. However, for large-scale use of these fibers, many investigations are still required. Indeed, although these fibers exhibit great advantages that play a role in their utilization, many other aspects that may prove determinant are still to be investigated. Among these, it is necessary to consider the aspects related to the possible effects of fiber degradation, specifically the lignin, which over time could lead to a reduction of the heavy metals removal capacity. All this must be related to the long-term behavior of other reactive substances, such as ZVI.

## Figures and Tables

**Table 1:** Values of heavy metal concentrations used for the batch tests and corresponding threshold limit values set by the Italian legislation (Legislative Decree 152/2006).

Heavy Metals	Concentrations Used for the Batch Tests (mg/L)	Threshold Limit Values of the Italian Legislation (Legislative Decree 152/2006) (mg/L)
Cu	43.00	1.000
Zn	65.00	3.000
Cd	0.05	0.005
Pb	0.17	0.010

**Table 2:** Removal percentage, final concentration and contact time values obtained by batch tests for cabuya fibers.

Heavy Metals	Removal Percentage (%)		Final Concentration (mg/L)		Contact Time (Hours)	
	Cabuya Fibers	ZVI	Cabuya Fibers	ZVI	Cabuya Fibers	ZVI
Copper (Cu)	99.24	93.42	0.32	2.83	14	24
Zinc (Zn)	90.09	94.17	6.44	3.81	24	14
Cadmium (Cd)	96.60	87.83	0.0017	0.0061	30	30
Lead (Pb)	100.00	100.00	0.00	0.00	22	30

**Table 3:** Results of the column tests for cabuya samples and for solutions of copper, zinc and cadmium.

Heavy Metals	Cu			Zn			Cd		
Column length (cm)	15	30	60	15	30	60	15	30	60
Initial concentrations (mg/L)	43.00	43.00	43.00	65.00	65.00	65.00	0.05	0.05	0.05
Min concentration *C_out_*(min) (mg/L)	0.118	0.000	0.000	1.110	1.050	0.515	4.49 × 10^−4^	5.82 × 10^−5^	3.48 × 10^−4^
Removal percentage (%)	99.72	100	100	98.29	98.39	99.21	99.10	99.88	99.30
Filtration time for *C_out_*(min)(h)	26	26	26	26	2	10	10	18	22
Duration of the release phase (h)	105	124	90	124	142	108	108	108	124

**Table 4:** Parameters *a* and *b* which define the Equation (5) for Cu, Zn and Cd.

Heavy Metals	*a*	*b*	R^2^
Copper	99.222	0.002	0.75
Zinc	96.400	0.0067	0.83
Cadmium	98.936	0.0015	0.06

**Table 5:** Average values of the constants of degradation for the heavy metal considered.

*t* (h)	*λ* (m^3^/kg day)		
	Copper	Zinc	Cadmium
0.25	0.206	0.422	0.191
0.5	0.487	0.532	0.297
1	0.927	0.749	0.658

**Table 6:** Parameters *c* and *d* defining the Equation (6) for Cu, Zn and Cd.

Heavy Metals	*c*	*d*	R^2^
Copper	0.0113	1.085	0.993
Zinc	0.1351	0.4139	0.988
Cadmium	0.0161	0.8923	0.973
